# Secretory IgA impacts the microbiota density in the human nose

**DOI:** 10.1186/s40168-023-01675-y

**Published:** 2023-10-21

**Authors:** Rob van Dalen, Ahmed M. A. Elsherbini, Mareike Harms, Svenja Alber, Regine Stemmler, Andreas Peschel

**Affiliations:** 1 Interfaculty Institute of Microbiology and Infection Medicine, Department of Infection Biology, University of Tübingen, Tübingen, Germany; 2 Cluster of Excellence EXC2124 Controlling Microbes to Fight Infections, University of Tübingen, Tübingen, Germany; 3 Present Address: Department of Medical Microbiology and Infection Prevention, Amsterdam UMC, University of Amsterdam, Amsterdam, The Netherlands; 4 German Center for Infection Research (DZIF), Partner Site Tübingen, Tübingen, Germany

**Keywords:** Mucosal immunology, Secretory IgA, IgA-seq, Nasal antibodies, Nasal microbiota

## Abstract

**Background:**

Respiratory mucosal host defense relies on the production of secretory IgA (sIgA) antibodies, but we currently lack a fundamental understanding of how sIgA is induced by contact with microbes and how such immune responses may vary between humans. Defense of the nasal mucosal barrier through sIgA is critical to protect from infection and to maintain homeostasis of the microbiome, which influences respiratory disorders and hosts opportunistic pathogens.

**Methods:**

We applied IgA-seq analysis to nasal microbiota samples from male and female healthy volunteers, to identify which bacterial genera and species are targeted by sIgA on the level of the individual host. Furthermore, we used nasal sIgA from the same individuals in sIgA deposition experiments to validate the IgA-seq outcomes.

**Conclusions:**

We observed that the amount of sIgA secreted into the nasal mucosa by the host varied substantially and was negatively correlated with the bacterial density, suggesting that nasal sIgA limits the overall bacterial capacity to colonize. The interaction between mucosal sIgA antibodies and the nasal microbiota was highly individual with no obvious differences between potentially invasive and non-invasive bacterial species. Importantly, we could show that for the clinically relevant opportunistic pathogen and frequent nasal resident *Staphylococcus aureus*, sIgA reactivity was in part the result of epitope-independent interaction of sIgA with the antibody-binding protein SpA through binding of sIgA Fab regions. This study thereby offers a first comprehensive insight into the targeting of the nasal microbiota by sIgA antibodies. It thereby helps to better understand the shaping and homeostasis of the nasal microbiome by the host and may guide the development of effective mucosal vaccines against bacterial pathogens.

Video Abstract

**Supplementary Information:**

The online version contains supplementary material available at 10.1186/s40168-023-01675-y.

## Background

The human nasal microbiome is an important factor in health and disease. Its composition is associated with respiratory disorders, such as chronic rhinosinusitis and allergies, and it can additionally host opportunistic pathogens such as *Staphylococcus aureus* [1, 2]. Compared to the gut microbiome, the nasal microbial community is relatively scarce and low in diversity [3]. The community composition varies strongly between individuals but can be divided into seven general profiles. These community state types (CSTs) are defined based on the presence of several hallmark members of the community, like *S. aureus*, *Staphylococcus epidermidis*, *Cutibacterium* spp., or *Corynebacterium* spp. [4]. Due to the presence of opportunistic pathogens in the nose, the host needs to protect this vulnerable mucosal barrier from infection and maintain homeostasis of the local microbiota. It can achieve this by several means, including limitation of nutrient availability in the nasal cavity in a process known as nutritional immunity [5], and through the production of antimicrobial defense proteins and peptides such as lysozyme, lactoferrin, and α- and β-defensins [6, 7]. Another hallmark feature of mucosal tissues, including the nasal mucosa, is the production of secretory IgA (sIgA) antibodies [8].

sIgA is a dimeric antibody that is highly abundant in nasal secretion, saliva, sweat, gut fluid, tears, and milk and is distinctively different from the monomeric IgA found in human serum [8]. It contributes to the defense of the mucosa through various mechanisms. For instance, sIgA can prevent the interaction of pathogens with the epithelium by agglutinating them and blocking their adhesion molecules, in a process known as immune exclusion [8]. This was observed in the gastrointestinal tract, in which sIgA coated and facilitated clearance of gut bacteria that are associated with the onset of colitis, thereby providing protection against disease [9]. Paradoxically, sIgA can also facilitate bacterial colonization of the mucosa by enhancing the mucus-binding properties of the bacterial cell surface through sIgA coating, such as is the case for the prominent human gut commensal *Bacteroides fragilis* [10, 11]. These contrasting effects of sIgA coating of bacteria are mediated by various factors, including the mucus flow rate, underlying pathology, and the affection of bacterial physiological processes [11, 12]. These important insights into the role of sIgA in the control of the microbiota were gained through studies on sIgA particularly in the gastrointestinal tract. Especially the development of the IgA‐seq technology, in which bacterial cells are sorted based on their sIgA-coating status and subsequently analyzed by metagenomic sequencing [9, 13, 14], has significantly contributed to our understanding of the role of sIgA in the homeostasis of the microbiome and defense against pathogens in the gastrointestinal tract [9, 10, 13, 15].

However, we currently still lack a comprehensive understanding of how sIgA affects the microbiota in other mucosal niches, in particular the human nose. The role of sIgA in the control of the nasal microbiota is implied by individuals with selective IgA deficiency suffering frequently from allergies and recurrent respiratory infections [16, 17]. Moreover, various respiratory pathogens produce immune evasion factors that inhibit sIgA, including an IgA serine protease produced by *Haemophilus influenzae,* the IgA-binding protein SSL7 secreted by *S. aureus*, and the secreted lambda-chain binding protein L from *Finegoldia magna* [18–22]. This suggests a benefit for these species to evade sIgA immune responses and thereby a role for sIgA in the defense against these pathogens.

*S. aureus* is of particular interest in this regard, as it is part of the normal human nasal microbiota, colonizing approximately one-third of the human population permanently and one-third intermittently [23]. However, nasal colonization by *S. aureus* is also an important risk factor for life-threatening infections [24]. Evasion of antibody responses is an important virulence strategy of *S. aureus*. In addition to the aforementioned secreted protein SSL7, it produces surface proteins staphylococcal protein A (SpA) and the second staphylococcal immunoglobulin-binding protein (Sbi) that bind various classes of antibodies. SpA is well-characterized to bind the Fc region of most human antibody classes, although not that of IgA. Through different binding sites, SpA also binds Fab domains of antibodies that belong to the VH3 family [25, 26]. To our knowledge, the interaction between SpA and VH3 Fab of IgA has not been reported, although the structure of the sIgA molecule does not preclude this interaction from taking place. Sbi, on the other hand, does not interact with sIgA as it only binds the Fc region of the IgG antibody class [27, 28]. Although the role of *S. aureus* antibody-binding proteins in invasive disease is well-studied, their potential role in the context of colonization is currently still unknown.

In this study, we aimed to determine whether sIgA affects the nasal microbiota density and composition. We therefore applied IgA-seq on nasal microbiota samples from male and female healthy adult volunteers to identify which bacterial species are targeted by sIgA, with a particular interest in *S. aureus*.

## Methods

### Human sample collection and processing

From each study participant, we sampled both anterior nares using E-Swabs (Copan Diagnostics). To ensure sample-to-sample reproducibility, all samples were collected by the same researcher following the same procedure for each sample: swabs were briefly dipped into sterile PBS (Lonza), swirled 10 times around in each nostril at a depth of 1–2 cm, and stored immediately in 1 ml of the provided Amies transport medium at 4 °C for up to 18 h, according to the manufacturer’s instructions.

Bacteria were eluted from the swab, centrifuged (1 min, 10,000 × *g*), aspirated, and resuspended in 1 ml PBS. Per sample, a 50-μl aliquot was taken as unstained control for fluorescence-activated cell sorting (FACS) and a 200-μl aliquot was kept at 4 °C for up to 4 h as a non-sorted control, until cell sorting was finished. The remainder was stained using FITC-conjugated F(ab’)2-Goat anti-human IgA (Invitrogen; 1/1000) in PBS + 0.1% BSA (Fraction V; Roth) for 20 min at 4 °C, washed once and resuspended in 100 μl PBS. All bacteria contained in the sample were subsequently sorted into FITC-positive and FITC-negative fractions, based on the FSC-A and anti-IgA-FITC parameters (Fig. 2B), using an MA900 cell sorter (Sony) at the Flow Cytometry Core Facility Tübingen, keeping the samples and the derived sorted fractions at 4 °C as much as possible throughout the sorting procedure.

Nasal sIgA was obtained from 1 ml nasal swab eluates by centrifugation (1 min, 10,000 × *g*) and sterile-filtration of the supernatant through a 0.4-μm pore (Merck). We determined sIgA concentrations by ELISA (Human IgA ELISA kit; Thermo Fisher), replacing the provided monomeric IgA standards with dimeric sIgA ELISA standards (Abnova). All samples were stored at – 20 °C until use.

### DNA extraction, amplification, and sequencing

We extracted DNA from the FITC-positive, FITC-negative, and non-sorted fractions immediately after cell sorting using the QIAmp DNA Microbiome kit (Qiagen) and a FastPrep-24 Classic homogenizer (MP Biomedicals) for mechanical lysis of the bacterial cells, according to the manufacturers’ specifications. For each run, a DNA extraction control was included, which was later verified to not contain any contaminations by 16S rRNA gene copy number quantification by qPCR. DNA was eluted in 50 μl of the supplied elution buffer, dried using a miVac centrifugal vacuum concentrator (SP Genevac), resuspended in 20 μl nuclease-free water (Ambion), and stored at – 20 °C until further use.

DNA amplification, amplicon library pooling, and 16S rRNA gene sequencing were performed by the NGS Competence Center Tübingen (NCCT). Specifically, the V1–V3 regions of the 16S rRNA gene were amplified according to Escapa et al. [29], using primers 518F and 27R (Table S2). The pooled amplicon library was sequenced on the Illumina MiSeq platform using MiSeq Reagent Kit v3 (Illumina).

### 16S rRNA gene copy number quantification

In addition to using total FACS event counts, nasal bacterial density was measured using a broad-coverage 16S rRNA gene qPCR assay described previously by Liu et al. [4, 30], with some modifications. Briefly, each non-sorted sample and DNA extraction control was amplified in 10 μl reactions in a 384-well PCR plate (Framestar) on a CFX384 Touch Real-Time PCR System (Bio-Rad), using the LightCycler 480 SYBR Green I Master qPCR kit (Roche) and 1.8 mM of each of the 16S rRNA gene primers 10 and 11 (Table S2) described previously for the BactQuant assay [30]. The used thermocycling parameters were 10 min at 95 °C for Taq activation, followed by 40 cycles of 15 s at 95 °C for denaturation, 1 min at 60 °C for annealing, and 20 s at 72 °C for extension, followed by a melting curve to inspect amplification of single products. An inrun standard curve was generated by PCR amplification of the respective region of the *Staphylococcus aureus* USA300 NRS384 16S rRNA gene, using GoTaq G2 Flexi DNA Polymerase (Promega) with 1 mM MgCl_2_, 0.4 mM of each dNTP and 0.2 μM of primers 12 and 13 (Table S2). The resulting single 505 bp amplicon was purified using a GeneJet PCR purification kit (Thermo Fisher), quantified by Qubit (Invitrogen), and diluted to 10^8^–10^2^ copies/reaction in serial tenfold dilutions to create the standard curve. All samples, DNA extraction controls, and standards were measured in the qPCR assay in triplicates. Automatic baseline and Ct threshold were set by the CFX Maestro software (Bio-Rad). None of the DNA extraction controls reached the threshold, indicating no detectable contamination was present. 16S rRNA gene copy numbers of the nasal DNA samples were calculated by interpolation of the standard curve of the Ct values against the log-transformed copy numbers.

### Data processing

Demultiplexed reads were checked for primer presence using Cutadapt (v1.18) [31]. We then used the DADA2 pipeline (v1.22.0) [32] in R (v4.1.3) for raw reads quality filtering and trimming, error rate learning, sample inference, pair concatenation, ASV calling, and chimera removal. The default parameters were used throughout, except for minParentAbundance = 15 and minFoldParentOverAbundance = 4 in the chimera removal step. The exported ASV table was imported in QIIME2 (v2021.11) [33] as a biom-formatted feature table. Taxonomic assignment was performed using a Naive-Bayes classifier trained as described previously on the eHOMD database (v15.1) [3, 29].

**Fig. 1:**
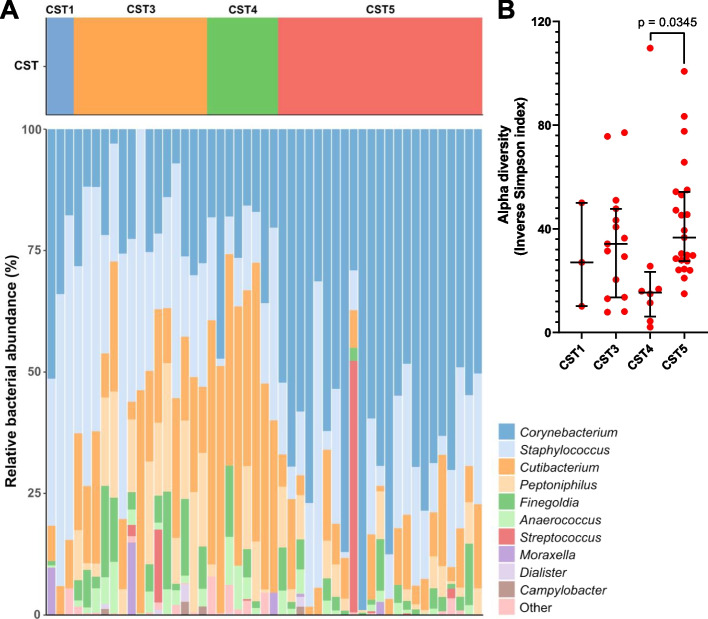
CST classification of the study population. Study participants were classified into the previously described CSTs, based on the non-sorted microbiota samples. **A** Relative abundance of the ten most common genera, stratified by CST. **B** Alpha diversity expressed as the Inverse Simpson index of all identified CSTs. Statistical differences in the alpha diversity were calculated by Kruskal–Wallis test with Dunn’s test for multiple comparisons

To calculate core diversity metrics, alpha-rarefaction curves were conducted in QIIME2. Then, using a sequence depth of 1100 reads (for CST classification and general community description, based on the non-sorted samples only; Fig. 1) or 800 reads (for computation of the IgA scores, based on the non-sorted as well as sorted fractions; Figs. 3, 4, and 5), keeping the sequence depth consistent for all samples per analysis. Matrices were computed using the Phyloseq (v1.38) [34] and Microbial (v0.0.20) [35] packages in R. CST classification was performed based on hierarchical clustering of the relative abundances, as described by Liu et al. [4]. IgA probability ratios were calculated using the IgAScores package in R [14].

### Bacterial strains and growth conditions

All used bacterial isolates are listed in Table S1. *S*. *aureus* (JE2 [36] and 35-1 (an in-house nasal isolate)), *S*. *epidermidis* [5], and *S*. *lugdunensis* [37] isolates were grown under aerobic conditions overnight at 37 °C with agitation in 5 ml tryptone soy broth (TSB; Oxoid). *C*. *accolens* and *C*. *simulans* isolates [38] were grown under anaerobic conditions for 42 h at 37 °C with agitation in brain-hearth infusion broth (BHI; Roth) with 0.4% Tween-80 (Sigma). *C*. *acnes* [38] was grown under anaerobic conditions for 42 h at 37 °C on basic medium-blood agar (BM-blood; 10 g/l soy peptone A3SC (Organo Technie), 5 g/l yeast extract (Ohly), 5 g/l NaCl (Merck), 1 g/l K_2_HPO_4_-trihydrate (Thermo Fisher), 1 g/l D-glucose-monohydrate (Sigma-Aldrich), 5% defibrinated sheep blood (Oxoid), 1.5% agar (BD Biosciences)), from which bacterial cells were collected prior to experiments. To simulate nutrient-limited conditions, *S. aureus* and *C. simulans* were grown as described above in 10% Todd-Hewitt broth (THB; Oxoid) [39, 40].

### Bacterial staining and flow cytometry

Bacteria were collected from triplicate cultures by centrifugation (1 min, 10,000 × *g*) and resuspended at OD_600_ = 0.4 in PBS with 0.1% BSA. In the case of *C. acnes*, bacterial cells were collected from agar plate using a sterile loop, resuspended in PBS with 0.1% BSA, and diluted to OD_600_ = 0.4. Bacteria were mixed 1:1 with sterile-filtered human nasal eluates diluted in PBS + 0.1% BSA to a final concentration of 0.3 μg/ml (for *S. aureus*) or 10 μg/ml sIgA (for all other species) and incubated for 30 min at 4 °C. The samples were subsequently washed in PBS + 0.1% BSA, stained using FITC-conjugated F(ab’)2-Goat anti-human IgA (Invitrogen; 1/1000) in PBS + 0.1% BSA for 20 min, washed again and fixed with 1% formaldehyde (Sigma) in PBS. After 15 min, formaldehyde was washed off and the samples were resuspended in PBS. Per sample, 10,000 events were acquired on an LSRFortessa X-20 flow cytometer (BD Biosciences) and analyzed using FlowJo 10 software (BD Biosciences). The acquired geomean fluorescence intensities (FI) were normalized to the matching unstained control to facilitate comparison between the different species.

### Construction of *S. aureus* USA300 JE2 SpA-AA

The nucleotide sequence of the *S. aureus* USA300 FPR3757 *spa* gene (GenBank locus tag SAUSA300_RS00585) was used as a reference to create *spa-AA*, in which the D70A, D71A, D131A, D132A, D189A, D190A, D247A, D248A, D305A, and D306A mutations were introduced [26]. Additionally, we used alternative codons for residues 56 to 63 to introduce a unique primer annealing site for screening purposes. As the 293 bp upstream region of the *spa* start codon until the *spa* stop codon was too high in complexity due to highly repetitive sequences, we had the region synthesized as two HiFi gBlocks (IDT), separated at the unique HindIII restriction site in the SpA A-domain. This reduced the template complexity of the first half sufficiently to synthesize it. For the second half, we introduced an additional 72 silent nucleotide substitutions to reduce the template complexity sufficiently to enable synthesis. Only frequently used codons (> 0.5%) were introduced, as listed in the Kazusa codon use database for *S. aureus* USA300 [41]. Sequences for all primers and gBlocks and primers are listed in Tables S2 and S3.

Both gBlocks were PCR amplified by Phusion Hot Start II polymerase (Thermo Fisher) using primers 1 and 2 (Biomers) for gBlock 1 and primers 3 and 4 for gBlock 2, digested at their unique HindIII (Thermo Fisher) restriction sites and ligated with T4 DNA ligase (Thermo Fisher), according to the manufacturers’ instructions. The ligated gBlock was subsequently digested and cloned into pIMAY [42] between the KpnI and SacI (Thermo Fisher) restriction sites, according to the manufacturers’ instructions, to create pIMAY-SpA-AA. This plasmid was transferred into *E. coli* IM08B [43] by heat shock procedure for plasmid amplification. Subsequently, *S. aureus* USA300 JE2 was transformed with pIMAY-SpA-AA and allelic exchange was performed as described by Monk et al. [42, 43]. Successful exchange of the wildtype *spa* allele with the variant *spa-AA* allele was verified by PCR using primers 5 and 6, and Sanger sequencing (Eurofins) of the locus using primers 5, 7, 8, and 9.

### Statistical analyses

The normality of the data was tested by Shapiro–Wilk test. Statistical differences between the two groups were tested by Mann–Whitney test, paired *t* test, or unpaired *t* test. Differences in alpha diversity of multiple groups were tested by the Kruskal–Wallis test with Dunn’s test for multiple comparisons. Correlations were analyzed by linear or non-linear regression analysis. IgA probability ratios were tested by Wilcoxon signed-rank test against a hypothetical value of 0. Significant differences are indicated by their exact *p* values. All statistical analyses were performed using GraphPad Prism (v9.3.1) or the R stats package.

## Results

### Study population and nasal community state types

Before analyzing the sIgA-binding capacities of nasal bacteria, we determined the overall nasal microbiota composition of the study participants in a cohort of 50 healthy human volunteers (Table 1), using the previously published method for V1–V3 16S rRNA gene sequencing that had been optimized for the human nasal microbiota [3, 29]. After the exclusion of one sample that failed to amplify before sequencing, all study participants were classified into the nasal CSTs (Fig. 1A) as described by Liu et al. [4]. Strikingly, we observed higher proportions of the *Corynebacterium* spp.-defined CST5 and *S. epidermidis*-defined CST3 in our study population compared to Liu et al. (46.9% vs. 20.0% for CST5; 30.6% vs. 22.5% for CST3) [4]. In contrast, the prevalence of the *S. aureus*-defined CST1 (6.1% vs. 12.4%)() and *Cutibacterium* spp.-defined CST4 (16.3% vs. 28.7%) were lower, and we found no Enterobacteriaceae-defined CST2, *Moraxella* spp.-defined CST6 or *Dolosigranulum pigrum*-defined CST7 in our study population. Consistent with the work of Liu et al. [4], we detected no differences in the microbiota composition between male and female study participants (Additional file 2: Figure S1). Of note, the alpha diversity of the highly prevalent CST5 was higher compared to that of CST4 (Fig. 1B), indicating that *Corynebacterium* spp. supports a broader microbial composition than *Cutibacterium* spp. In line with the findings of Escapa et al. [3], *Corynebacterium*, *Cutibacterium*, and *Staphylococcus* were the most prevalent and abundant genera in our study population. In contrast, we detected *Lawsonella clevelandensis* only in a single individual, despite it being described previously as a highly prevalent nasal microbiota member [3]. *S. aureus* was detected in 16% of the samples, making up between < 1% and 47% of the total reads in the respective samples (also see Fig. 4).

**Table 1:** Study population and sex differences

	*n* (%)	Age (years) [mean (range)]	sIgA concentration (μg/ml) [mean (range)]	Bacterial density (absolute number of FACS events per sample) [median (range)]	sIgA-positive fraction size (%) [median (range)]
All	50	37 (21–61)	158 (3–365)	78,900 (8270–264,395)	21.5 (0.6–47.0)
Male	17 (34%)	37 (25–58)	183 (3–365)	80,174 (36,023–264,395)	24.6 (2.4–47.0)
Female	33 (66%)	37 (21–61)	145 (12–349)	78,634 (8 270–218,403)	20.9 (0.6–45.7)
			*p* = 0.19	*p* = 0.53	*p* = 0.21

Statistical differences between male and female study participants were tested by Mann–Whitney test (for bacterial density and fraction size) or unpaired *t* test (for sIgA concentration)

### Nasal sIgA limits the nasal bacterial density

**Fig. 2:**
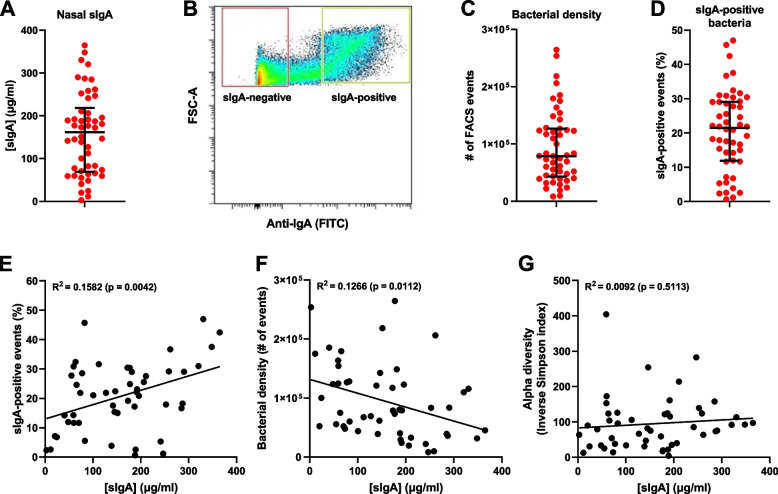
Nasal sIgA concentration and correlation with bacterial density and diversity. **A** Distribution with the median ± interquartile range of nasal sIgA concentrations in the filtered nasal eluates of all study participants. **B** Sorting by FACS of a representative nasal microbial sample into sIgA-positive (green gate) and sIgA-negative (red gate) fractions, based on forward scatter (FSC-A) and anti-IgA-FITC staining. **C**, **D** Distribution with the median ± interquartile range of **C** nasal bacterial density, expressed as the total number of events analyzed by FACS, and **D** percentage of sIgA-positive events detected by FACS. **E**–**G** Correlation of the nasal sIgA concentration with (**E**) the percentage of sIgA-positive events detected by FACS, (**F**) the bacterial density expressed as the total number of FACS events, and (**G**) the alpha diversity expressed as the Inverse Simpson index in the non-sorted fraction. Correlations were tested by linear regression analysis

We next determined the sIgA concentration of nasal swab eluates from all study participants. This showed a surprisingly large variation in sIgA quantity between individuals, ranging from 3 to 365 μg/ml (Fig. 2A). Since the swabs were eluted in a volume of 1 ml, these concentrations can be interpreted as the absolute quantities of sIgA collected using a single swab. To elucidate how human nasal sIgA targets nasal bacteria and affects microbiota diversity, bacteria obtained from human nasal swabs were stained with a fluorescently labeled antibody specific for human IgA, thereby labeling pre-deposited sIgA on the native microbiota samples. The bacteria were subsequently sorted by FACS according to their fluorescence into sIgA-positive and sIgA-negative fractions (Fig. 2B). We used the total FACS event count per swab as a measure for the absolute bacterial density (Fig. 2C) and determined the proportion of FACS events sorted into the sIgA-positive fraction (Fig. 2D). The nasal sIgA concentration showed a positive correlation with the percentage of sIgA-positive events (Fig. 2E). Importantly, we observed that the nasal sIgA concentration correlates negatively with the bacterial density (Fig. 2F), suggesting that sIgA coating of bacteria lowers the overall bacterial capacity to prevail in the nasal microbiome. As an additional measure of the nasal bacterial density, we quantified the 16S rRNA gene copy number per swab. This measure was highly correlated with the FACS event count (Additional file 3: Figure S2A), and similarly correlated negatively with the nasal sIgA concentration (Additional file 3: Figure S2B). However, the correlations between the bacterial density and the sIgA concentration do not consider any differences in sIgA coating between the taxa present or the subsequent functional effects of sIgA coating. Furthermore, we found no correlation between the sIgA concentration and the alpha diversity, indicating that sIgA did not impact the diversity of the nasal microbiota (Fig. 2G). Of note, no differences in the bacterial density, sIgA-positive fraction size, or sIgA concentration between males and females were identified (Table 1), in contrast to a previous report of a higher nasal bacterial density in males [4]. Taken together, these findings indicate that sIgA limits the overall nasal bacterial density but not the diversity.

### sIgA targeting of nasal microbiota members is highly individual

**Fig. 3:**
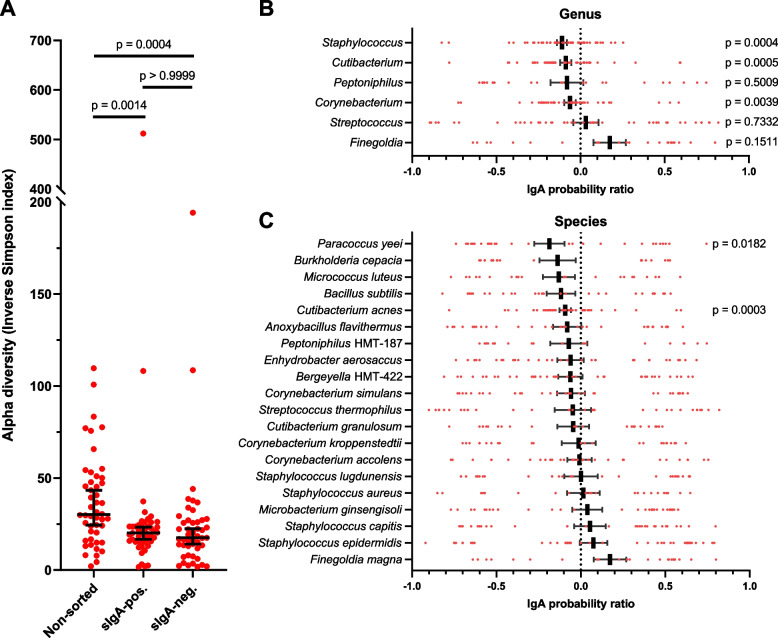
sIgA targeting of nasal bacteria. **A** Alpha diversity of the non-sorted, sIgA-positive and sIgA-negative fractions, expressed as the Inverse Simpson index. Individual values are depicted in red, with the median and 95% confidence interval in black. Statistical differences were calculated by Kruskal–Wallis test with Dunn’s test for multiple comparisons. **B**, **C** sIgA targeting is presented as the IgA probability ratio on a scale from − 1 to + 1 for (**B**) the top-6 most prevalent genera and (**C**) the top 20 most prevalent species (also see Figure S3). Individual values are depicted as red dots, with the mean ± SEM in black. IgA scores were tested by Wilcoxon signed-rank test against a hypothetical value of 0

To analyze whether nasal sIgA targets all nasal bacteria equally or tends to target different species in each host, the microbial composition of the sIgA-positive and sIgA-negative samples were determined as described above. After exclusion of one sample (CST1) for which 16S rRNA gene sequencing failed due to too low DNA yields in one of the sorted fractions, we observed that bacterial sorting based on sIgA coating resulted in a substantially altered sample composition compared to the non-sorted samples. In particular, the alpha diversity of both the sIgA-positive and sIgA-negative fractions was significantly reduced compared to the non-sorted control (Fig. 3A). For all genus-level and species-level taxa, we calculated the sIgA targeting efficacy, using the ‘IgA probability ratio’ (referred to as ‘IgA score’ from here on) defined by Jackson et al. [14] (Additional file 1). This score ranges from − 1 (all bacteria of a given taxon are in the sIgA-negative fraction) to + 1 (all bacteria of a given taxon are in the sIgA-positive fraction). Importantly, IgA scores can only be calculated for species that are present in the sample and will therefore not inform about absent species. Furthermore, the enrichment of species in the sIgA-positive or sIgA-negative fractions enhanced the detection of low-abundant species, allowing for the computation of IgA scores for species that were initially not detectable in the non-sorted samples. We made an arbitrary selection of the top 20 most prevalent species and top 6 most prevalent genera, based on the IgA scores, for further analysis (Additional file 4: Figure S3). For the majority of genus-level or species-level taxa, we observed a highly variable IgA score that in several taxa varied across virtually the entire range from − 1 to + 1 in the study population (Additional file 4: Fig. 3B, C). This distribution indicates highly variable sIgA responses to the microbiota by the individual hosts. On genus level, there was an overall trend towards negative IgA scores, with *Staphylococcus*, *Cutibacterium,* and *Corynebacterium* having IgA scores significantly below 0, indicating that these genera were generally not effectively targeted by nasal sIgA antibodies (Fig. 3B). On species level, we again observed an overall trend towards negative IgA scores, although only two species (*Cutibacterium acnes* and *Paracoccus yeei*) had IgA scores significantly below 0 (Fig. 3C). Interestingly, for the majority of species the IgA scores were not normally distributed across the IgA score range of − 1 to + 1 (Shapiro–Wilk normality test outcome of *p* < 0.05 in 17 out of 20 species), but instead formed three distinct clusters of negative, zero-centered, or positive IgA scores (Fig. 3C). This pattern could be observed for, e.g., *S. aureus*, *S. epidermidis,* and *Corynebacterium accolens*. Since every data point represents an individual host, this uneven IgA targeting efficacy indicates three different main modes of sIgA interactivity with these species across individuals, in which only some hosts produce an effective sIgA response against particular microbiota members, whereas others have intermediary responses or do not effectively target these microbiota members at all. Strikingly, other species such as *C. acnes*, lack this separation into three clear clusters and show a more consistently clustered distribution of IgA scores (Fig. 3C).

**Fig. 4:**
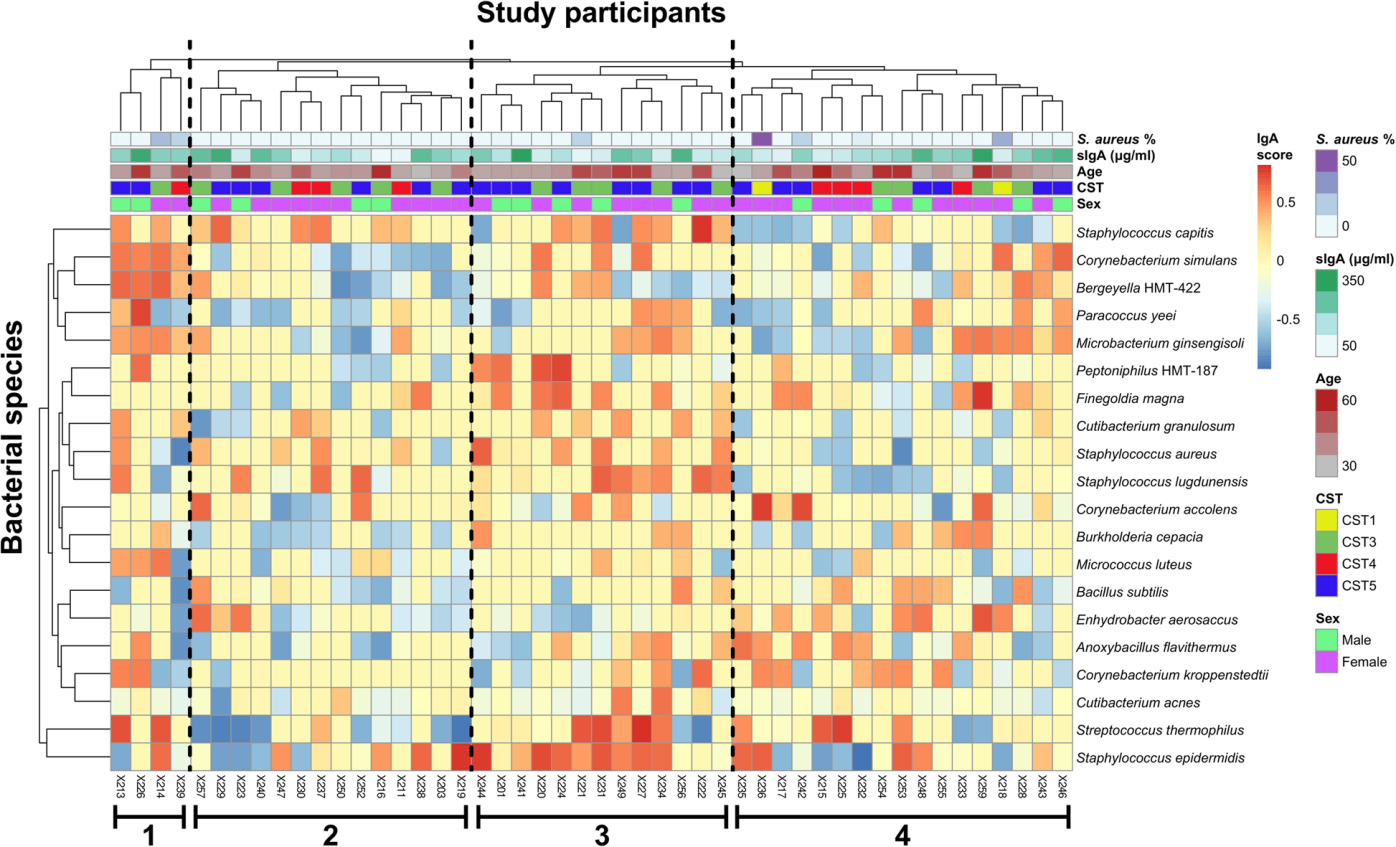
Hierarchical cluster analysis of the sIgA targeting. The study participants (columns) and the top-20 nasal bacterial species (rows) were clustered based on the IgA scores, with missing values (when no IgA score could be calculated due to the absence of the species) treated as 0. The proportion of *S. aureus* reads, sIgA concentration, and CST of the sample, as well as the age and sex of the respective study participant, are displayed at the top. Branches that contain hosts with distinct sIgA targeting profiles are indicated and numbered below. See Figure S4 for the same analysis expanded to all detected nasal bacterial species

**Fig. 5:**
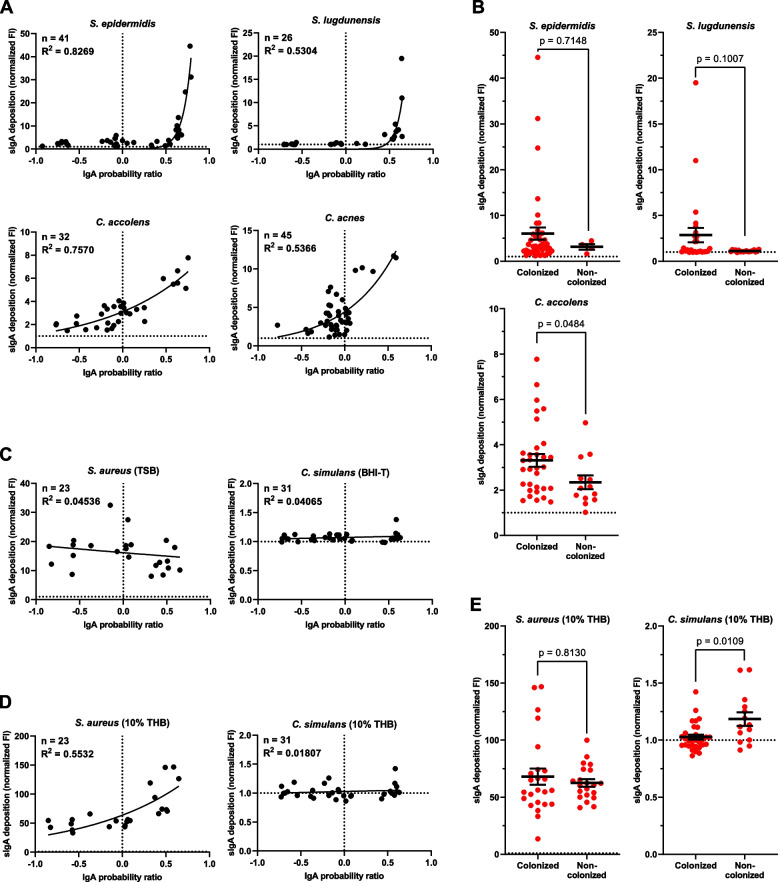
Validation of the IgA-seq outcomes.** A**,** C**,** D** Correlation between sIgA deposition from filtered and concentration-adjusted nasal eluates on cultured nasal isolated of a selection of bacterial species, as assessed by flow cytometry, and the IgA probability ratio calculated for the same species of the matching study participants. All strains were grown in nutrient-rich TSB (for *Staphylococcus* spp.) or BHI-Tween-80 (BHI-T; for *Corynebacterium* and *Cutibacterium* spp.) (**A**,** C**) or in nutrient-limiting 10% THB (**D**). All samples were normalized to 10 μg/ml sIgA, except for *S. aureus* 35-1 (0.3 μg/ml sIgA). Fluorescence intensity (FI) was measured by flow cytometry in triplicates and normalized to the corresponding unstained controls, with a fold change of one (no detectable deposition) being represented by a dashed horizontal line. Correlations were analyzed by non-linear regression. **B, E** sIgA deposition of nasal sIgA of individual study participants on nasal isolates of *S. epidermidis*, *S. lugdunensis,* and *C. accolens* grown in nutrient-rich conditions (**B**), and *S. aureus* 35-1 and *C. simulans* grown in nutrient-limiting conditions (10% THB) (**E**), stratified by colonization status. Statistical differences were tested by Mann–Whitney test

We next examined how sIgA targeting varied on the individual host level by hierarchical cluster analysis of the IgA scores of both the hosts and the previously selected top 20 species (Figs. 4 and 5). Based on this analysis, several clusters of hosts with distinct sIgA profiles could be distinguished. Branches 1 and 3 contained individuals who produce sIgA that broadly covers their nasal microbiota members, as indicated by the generally positive IgA scores of these individuals. In contrast, sIgA of the individuals in branch 2 was generally poorly reactive with the nasal microbiota members of these individuals, as indicated by the generally negative IgA scores. Branch 4 instead contained individuals with varying levels of sIgA reactivity to different species present. Importantly, sIgA targeting did not correlate with the *S. aureus* carrier status, nasal sIgA concentration, age, sex, or CST of the host, since no clustering of these factors with the IgA score was observed (Fig. 4). These results suggest a highly individualized interplay between our nasal microbiota and our immune system.

### Validation of sIgA-targeted nasal microbiota species

To validate the IgA-seq approach, we measured by flow cytometry sIgA deposition on in vitro cultured cells of a selection of representative nasal isolates from bacterial species that were highly prevalent in this study (Fig. 5). As sIgA source we used individual sterile-filtered nasal swab eluates collected from the study participants, adjusted to the same sIgA concentration. For each species analyzed this way, the signal of the deposited sIgA on the bacteria was subsequently plotted against the IgA score of the same species from the corresponding study participant. Using this method, we could validate the nasal IgA-seq approach, as we observed a correlation between the sIgA deposition and the IgA score for *S. epidermidis*, *Staphylococcus lugdunensis*, *C. accolens*, and *C. acnes* (Fig. 5A). In all cases, the individuals with the highest IgA scores also ranked among the highest for sIgA deposition. However, for all species, we also observed a loss of resolution in the sIgA deposition for individuals with IgA scores below 0.5. This is indicative of a lower sensitivity of the sIgA deposition assay, particularly in the case of samples with low IgA scores. Regardless, for these species, we could broadly validate the IgA-seq outcomes. We additionally measured sIgA deposition for the study participants for whom no IgA score could be calculated due to the absence of these species from their nasal microbiota (Fig. 5B). For *C. accolens* in particular, non-colonized individuals generally produced specific sIgA, although at significantly lower levels compared to colonized individuals. For *S. epidermidis* and *S. lugdunensis*, we observed a similar trend, with individuals not colonized by *S. lugdunensis* in particular producing hardly any specific sIgA (Fig. 5B). As all study participants were colonized by *C. acnes* (Additional file 4: Figure S3), the levels of *C. acnes*-specific nasal sIgA in non-colonized individuals remains unknown.

Two major exceptions from these observations were *S. aureus* and *Corynebacterium simulans*, for which no correlation between the IgA score and the sIgA deposition was found (Fig. 5C). In case of *S. aureus*, we were required to use a 30-fold lower sIgA concentration in the flow cytometric assay as a result of the universally high levels of *S. aureus*-reactive sIgA antibodies in the nasal swab eluates relative to all other tested species (Fig. 5A, C, note the scale of the y-axes). On the other hand, for *C. simulans* we could hardly detect any sIgA deposition at all. For both *S. aureus* and *C. simulans*, the lack of correlation between the IgA score and the sIgA deposition is potentially the result of large differences between the epitope repertoires produced by the representative strain isolates used in the sIgA deposition assay and the native strains colonizing the nares of the study participants. Alternatively, the nutrient-rich broths used for the in vitro cultivation of the isolates used in the sIgA deposition assay (TSB for *S. aureus*; BHI with 0.4% Tween-80 for *C. simulans*) could potentially induce different epitope repertoires compared to the nutrient-poor conditions encountered in the human nares. This would result in sIgA deposition profiles that are not representative of the natural niche and therefore be poorly comparable to the IgA scores of the native samples. To test this hypothesis, we grew the same *S. aureus* and *C. simulans* isolates in nutrient-limited 10% Todd Hewitt broth (THB), as described previously [39, 40]. Indeed, in stark contrast with *S. aureus* grown in nutrient-rich TSB, sIgA deposition on *S. aureus* grown in nutrient-limited 10% THB correlated with the IgA scores obtained by IgA-seq (Fig. 5D). This effect was specific for *S. aureus*, as sIgA deposition on *C. simulans* grown in nutrient-limited 10% THB was still virtually absent. This suggests that the *S. aureus*-reactive nasal sIgA antibodies are at least partially dependent on epitopes that strongly vary depending on the growth condition.

We additionally measured sIgA deposition on *S. aureus* and *C. simulans* (both grown in 10% THB) for the study participants for whom no IgA score could be calculated due to absence of these species from their nasal microbiota (Fig. 5E). Remarkably, nasal sIgA deposition from non-colonized individuals on *S. aureus* was generally high, at a similar level as that of *S. aureus*-colonized individuals. This finding suggests that nasal sIgA reactive with *S. aureus* is a universal phenomenon. Furthermore, it contradicts the *S. aureus* IgA scores (Fig. 3), which suggested that some individuals may not produce large amounts of sIgA reactive to *S. aureus*. In case of *C. simulans*, we instead measured significantly higher sIgA deposition in non-colonized individuals than in colonized individuals, although the actual deposition levels were still very minor, indicating a low degree of sIgA reactivity with *C. simulans* (Fig. 5E).

### *S. aureus* binds sIgA epitope-independently through SpA

**Fig. 6:**
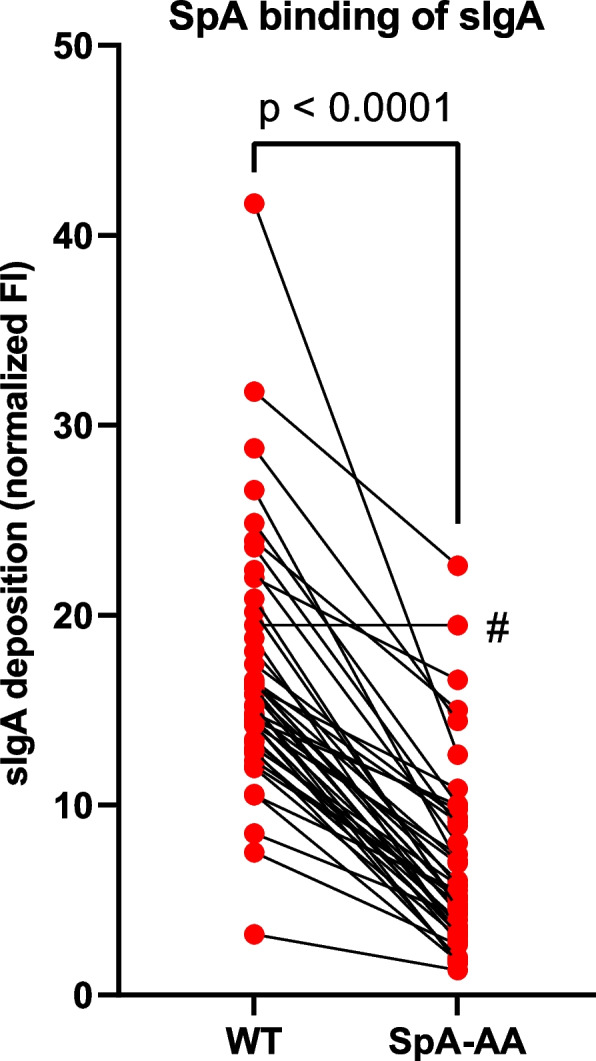
*S. aureus* binds sIgA epitope-independently via SpA. sIgA deposition of nasal sIgA of individual study participants (*n* = 46) on *S. aureus* USA300 JE2 wildtype (WT) and the SpA-AA mutant deficient for binding of antibody Fab regions. Statistical differences were tested by paired *t* test. A single sample (#) did not follow the trend of lower sIgA deposition on the SpA-AA mutant

In the case of *S. aureus*, the results obtained with the IgA-seq and sIgA deposition assays were in contradiction with each other. Since *S. aureus* is well-known to produce multiple antibody-binding proteins such as SpA, Sbi, and SSL7 [19, 26, 28], we hypothesized that the universal sIgA deposition on *S. aureus* observed in the sIgA deposition assay might not be epitope-based, but rather a result of epitope-independent binding of sIgA. Although unable to bind IgA-Fc, SpA was the most likely candidate to be this interaction partner, because it is a cell-wall-bound protein that has the potential to bind to the Fab region of a subset of the sIgA antibodies, namely those that belong to the VH3 structural family [25]. Using an *S. aureus* SpA-AA mutant that is deficient for VH3-Fab binding [26], we indeed observed a significant reduction in the deposition of sIgA (Fig. 6). This reduction could be observed for nasal sIgA from all but one of the study participants, indicating the presence of significant amounts of VH3 sIgA antibodies on the nasal mucosa. The expression of SpA by *S. aureus* in the human nares is known to be highly variable between individuals and often distinctly different from conditions used in vitro [44]. We therefore speculate that for the individuals with negative IgA scores but with substantial sIgA deposition in vitro, the colonizing *S. aureus* strains of these individuals produce SpA at only low levels in the nares. Overall, these data indicate that the deposition of nasal sIgA on *S. aureus* is largely dependent on epitope-independent SpA-sIgA interactions.

## Discussion

We currently lack a comprehensive understanding of how sIgA affects the microbiota and supports microbiome homeostasis and immune defense in the human nasal mucosa. To determine whether sIgA affects the nasal microbiota composition, we applied IgA-seq on nasal microbiota samples from healthy adults to identify which bacterial species are targeted by sIgA. We observed a strong negative correlation between the nasal sIgA quantity and the bacterial density, suggesting that sIgA limits the bacterial capacity to colonize, and a highly individualized interplay between mucosal sIgA antibodies and the nasal microbiota. Importantly, we showed that for the clinically relevant opportunistic pathogen *S. aureus*, sIgA reactivity was largely dependent on epitope-independent interaction with the antibody-binding protein SpA through the Fab region of the sIgA antibodies.

On the host side, individuals differed substantially in the quantity of nasal sIgA that is produced during healthy conditions (approximately 100-fold), with no differences between males and females. Similar ranges of nasal sIgA concentrations have been reported previously by others [45–47]. The reason behind this wide range remains elusive but could potentially be explained by differential antigenic exposure as well as the local conditions during which the immune system encounters the antigens. It seems likely that the quantity of secreted nasal sIgA under these baseline conditions may have consequences for colonization and disease susceptibility. When considering the whole community, high nasal sIgA amounts limited nasal bacterial density but not the microbial diversity. On the level of the individual species, IgA-seq revealed highly varying individual nasal sIgA profiles that were not dependent on age, sex, or CST of the host. Strikingly, for many species, such as *S. aureus*, *C. accolens* and *F. magna*, hosts fall into one of three distinct IgA score groups of negative, zero-centered or positive IgA scores. This hints at three main modes of sIgA induction by these species, in which only several individuals produce a clear sIgA response against particular microbiota members, whereas others do not target these microbiota members at all or only mildly. Importantly, through cluster analysis, we identified clusters of hosts producing an sIgA repertoire that broadly covers their nasal microbiota, whereas others are generally poorly reactive to their nasal microbiota or display varying levels of sIgA reactivity to different members. We therefore speculate that the sIgA response generally depends on the host’s genetics, immune system, and/or the local conditions (e.g., ongoing inflammation) during which the immune system encounters the bacteria, rather than the inherent immunogenicity of the colonizing bacterial species, implying a highly individualized interplay between the immune system and our microbiota. Furthermore, the levels of sIgA against a particular species also depend on the colonization status of these species in the microbiome that can thereby provide constant stimulation of the immune system, since sIgA deposition against the majority of tested species tended to be lower in non-colonized individuals than in colonized individuals. Currently, it is still challenging to define the role of sIgA in the nasal cavity. In the gastrointestinal environment, sIgA can both promote and attenuate bacterial colonization, depending on mucus flow rate, disease state, and differential adhesion between species [9–12]. For the nasal niche, an important next step in this regard would be to compare baseline sIgA targeting with that during diseased states, such as chronic rhinosinusitis, cystic fibrosis, antibiotic-induced dysbiosis, or other respiratory disorders. Furthermore, functional studies are required to define how sIgA coating affects bacteria in the nasal mucosa, taking into consideration the unique properties of this particular niche, such as the low mucus flow rate and high exposure to the environment.

On the bacterial side, we did not detect any species that is universally targeted by sIgA. Both invasive and non-invasive bacteria show reactivity with sIgA without a notable pattern across the tested hosts. Therefore, bacterial aggressiveness is seemingly not correlated to the induction of nasal sIgA antibody responses. A potential exception to this is *F. magna*, which showed the highest sIgA reactivity of any species or genus, though not significant in our study population. Although *F. magna* typically does not cause infection in healthy individuals, it is an opportunistic pathogen that can cause significant morbidity in immunocompromised hosts or hosts with barrier disruptions, ranging from skin abscesses to bone and prosthetic joint infections [48, 49]. Additionally, it has the potential to activate neutrophils and trigger NETosis [50]. These invasive features could explain the high sIgA reactivity to *F. magna*. Alternatively, the observed sIgA targeting could be partially or fully mediated by the *F. magna* antibody-binding Protein L and therefore be epitope-independent [21, 22]. In the case of *S. aureus*, we confirmed epitope-independent interactions to contribute substantially to nasal sIgA binding. *S. aureus* showed high deposition of sIgA from all study participants, despite not being universally sIgA-targeted in the IgA-seq approach. We determined that this high level of interaction was largely caused by the epitope-independent interaction of antibody-binding protein SpA with sIgA. Since SpA cannot bind IgA Fc, this interaction was dependent on the Fab-binding of VH3-family sIgA [26]. SpA involvement in this phenomenon was further supported by the increase in sIgA deposition of *S. aureus* in nutrient-limited growth conditions, during which there is a lack of *spa* repression by the *agr* quorum sensing system [44] and thus an increase in SpA production. Importantly, *spa* expression in the human nares is highly variable between individuals and significantly different from *spa* expression by the same strains in vitro [44]. These findings provide a potential explanation for the negative *S. aureus* IgA scores we observed in multiple individuals, despite the sIgA of all individuals showing the potential to deposit on the *S. aureus* cell surface under laboratory conditions. However, further studies are required to fully functionally characterize SpA-mediated binding of sIgA Fab in situ. Additionally, the functional consequences of SpA-mediated sIgA coating of *S. aureus* in the human nasal mucosa are currently still unknown. Based on the function of sIgA in the gastrointestinal tract [11], these could range from *S. aureus* clearance from the nasal cavity to supporting colonization through enhanced mucus- or epithelial interactions. Intentionally coating itself in host immune factors to gain benefit would not be an uncommon strategy for *S. aureus*, which is known to cover itself with immune factors, such as fibrinogen through the Efb protein [51]. Further research on the functional consequences of SpA-sIgA interaction on the human nasal mucosa is therefore necessary.

The classification of nasal microbial community compositions into CSTs provides an easy label to describe a particular microbial community, based on key indicator genera or species [4]. The nasal microbiota compositions of our study population were generally in line with previously published data on healthy individuals [3, 4]. However, we observed several striking differences in CST prevalences. In particular, none of the analyzed microbiota classified as the *M. catarrhalis*-characterized CST6, *D. pigrum*-characterized CST7, or Enterobacteriaceae-characterized CST2, as these taxa were very rare in our study population. In contrast, our study population featured a large proportion of the *Corynebacterium*-characterized CST5. A similar absence of CST6 and CST7 and a large proportion of CST5 was also reported in a study on a population of workers and visitors of pig farm [52]. Although our study population had no relation to pig farming, it seems plausible that certain environmental or occupational settings have an impact on the composition of the nasal microbiota and thereby affect CST prevalences. Furthermore, in our study, we sequenced the V1–V3 region of the 16S rRNA gene, which offers enhanced resolution in the nasal environment [29], instead of the V3–V4 region that has been used in other nasal microbiota studies that use CST classification [4, 52]. This methodological difference may have affected the outcome of the taxonomical classification and thereby also of the CST classification. Finally, similar to the human gut microbiota enterotypes, CSTs represent arbitrary groupings that imperfectly capture the compositional variation of nasal microbiota communities, and might therefore be too reductive in nature, as well as poorly comparable between studies and study populations.

In this work, we quantified nasal bacterial density using both particle counting by FACS as well as the measurement of the 16S rRNA gene copy number by qPCR. These two measures of bacterial density were highly correlated, but the qPCR method yielded 20- to 70-fold higher density estimates, depending on the sample. This discrepancy is presumably the result of multiple 16S rRNA gene copies per genome [4] as well as an underestimation of the density by particle counting due to naturally occurring bacterial aggregates. Our qPCR-based nasal bacterial density estimates are in line with those of Liu et al., with an average density of 10^6^–10^7^ 16S rRNA gene copies per swab [4].

Our results underline the importance of laboratory testing of bacteria in their native niche or in conditions that mimic their niche environment. The use of nutrient-rich culture media in general research practice will often hide phenomena that depend on the unique conditions in the body niches that these bacteria grown in, broadly affecting regulatory mechanisms, metabolic adaptation, and expression of epitopes [5, 44, 53]. Measuring microbial processes or host-microbe interaction in the native state in their niche, such as by IgA-seq, is therefore essential to better understand colonization, infection, and microbiome homeostasis. IgA-seq has additionally hinted at the potential significance of low-abundant nasal species, such as *Paracoccus yeei*. Furthermore, sIgA-based enrichment of microbiota allowed for the detection of low-abundant species that would otherwise not have reached the detection limit in the current 16S rRNA gene sequencing method and associated bioinformatical pipelines. We therefore propose that the general strategy of microbial enrichment based on bacterial properties or surface molecules can be used to enhance sequencing-based detection of low-abundant species.

The nasal IgA-seq approach revealed highly individualized interplay between mucosal sIgA antibodies and the nasal microbiota. The results of this study are highly relevant in the context of nasal mucosal vaccination strategies. Mucosal vaccines are favorable in their capacity to induce robust protective immune responses at the initial site of infection [54, 55]. For instance, SARS-CoV-2 nasal vaccination is a promising strategy to induce respiratory mucosal immunity against the virus, where intramuscular vaccines fall short [56]. Also against bacterial respiratory pathogens there is a great unmet need for mucosal vaccines, including for *S. aureus*, *Mycobacterium tuberculosis*, *Streptococcus pneumoniae*, *Bordetella pertussis*, and *H. influenzae* [55]. The first comprehensive insight into the targeting of the nasal microbiota by sIgA antibodies that this study has offered can aid a better understanding of the shaping and homeostasis of a healthy nasal microbiome by the host’s immune system and offers potential leads for intervention in disease‐associated microbiota members.

## Conclusions

We used IgA-seq on nasal microbiota samples from healthy adults to identify which bacterial species are targeted by sIgA. This revealed a highly individualized interplay between nasal sIgA antibodies and the local microbiota. Importantly, the nasal sIgA quantity and the nasal density were negatively correlated, suggesting that sIgA limits the bacterial capacity to colonize in the human nose. In the case of the clinically relevant opportunistic pathogen *S. aureus*, sIgA reactivity was largely dependent on epitope-independent interaction with the antibody-binding protein SpA through the Fab region of the sIgA antibodies.

### Supplementary Information

**Additional file 1.** IgA scores of all genera and species in the dataset.

**Additional file 2:** **Figure S1.** Compositional analysis by sex. Relative abundance of the ten most prevalent genera, stratified by sex.

**Additional file 3:** **Figure S2.** Nasal bacterial density, quantified by 16S rRNA gene qPCR. **A** Correlation of the log-transformed 16S rRNA gene copy numbers with the log-transformed total event count measured by FACS (also see Fig. 2C). **B** Correlation of the nasal sIgA concentration with the bacterial density expressed as the 16S rRNA gene copy number. Correlations were tested by linear regression analysis.

**Additional file 4:** **Figure S3.** Top-20 most prevalent species, based on IgA scores. The frequency indicates in which percentage of the samples an IgA score for a given species could be calculated.

**Additional file 5:** **Figure S4.** Hierarchical cluster analysis of the sIgA targeting. The study participants (columns) and the top-20 nasal bacterial species (rows) were clustered based on the IgA scores, with missing values (when no IgA score could be calculated due to absence of the species) treated as 0. The proportion of *S. aureus* reads, sIgA concentration and CST of the sample, as well as the age and sex of the respective study participant, are displayed at the top. Related to Fig. 4.

**Additional file 6:** **Table S1.** Bacterial strains used in this study.

**Additional file 7: Table S2.** Primers used in this study.

**Additional file 8: Table S3.** HiFi gBlocks used in this study.

## Data Availability

The dataset supporting the conclusions of this article is available from NCBI SRA under BioProject PRJNA923578, https://www.ncbi.nlm.nih.gov/bioproject/923578. The metadata and code used in R and QIIME2 are available on GitHub, https://github.com/AhmedElsherbini/Code-for-RVD-et-al.-2023.
