# Supplementary material for: Secretory IgA impacts the microbiota density in the human nose
Source: Microbiome. 2023 Oct 21;11:233. doi: 10.1186/s40168-023-01675-y (PMC10589987; doi:10.1186/s40168-023-01675-y)
Supplement: Supplementary file 3 — Additional file 2: Figure S1. Compositional analysis by sex. Relative abundance of the ten most prevalent genera, stratified by sex. [file 40168_2023_1675_MOESM2_ESM.pdf]

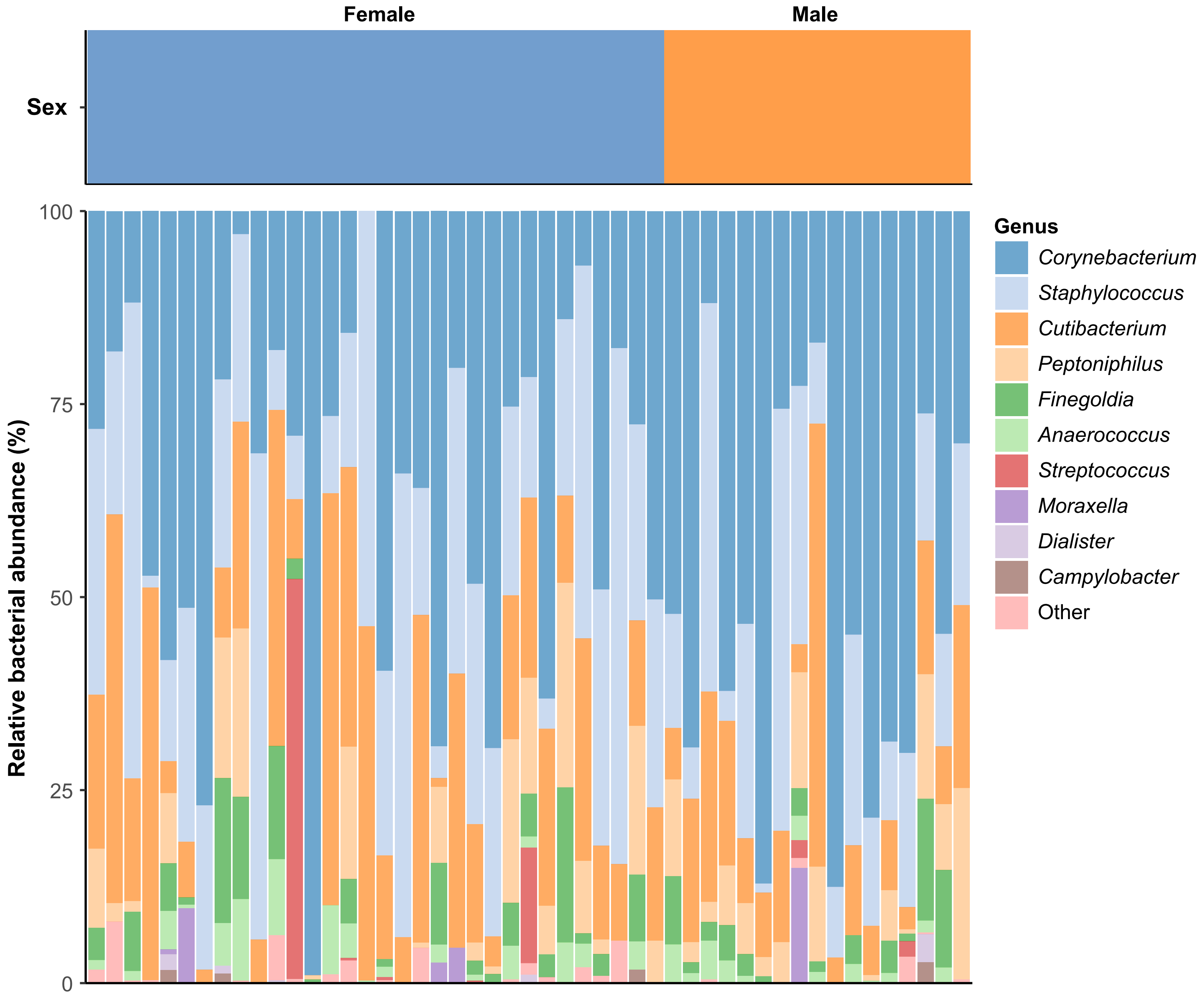

Female
Male
Sex
100
75
50
25
0
Relative bacterial abundance (%)
Genus
Corynebacterium
Staphylococcus
Cutibacterium
Peptoniphilus
Finegoldia
Anaerococcus
Streptococcus
Moraxella
Dialister
Campylobacter
Other
