# Supplementary figures and images for: Secretory IgA impacts the microbiota density in the human nose
Source: Microbiome. 2023 Oct 21;11:233. doi: 10.1186/s40168-023-01675-y (PMC10589987; doi:10.1186/s40168-023-01675-y)

**A**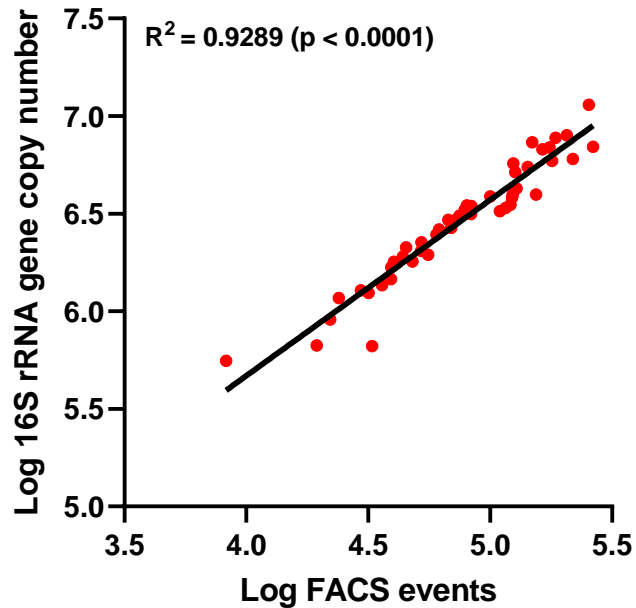**B**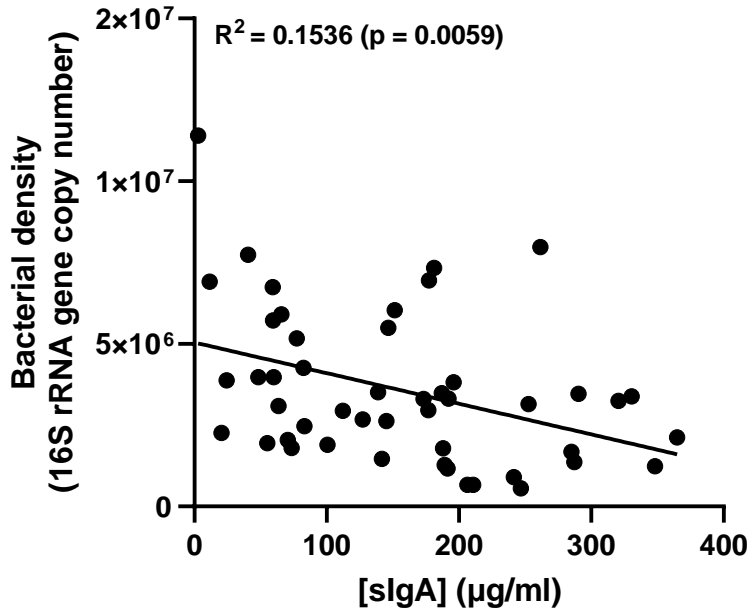

Supplement: Supplementary file 4 — Additional file 3: Figure S2. Nasal bacterial density, quantified by 16S rRNA gene qPCR. A Correlation of the log-transformed 16S rRNA gene copy numbers with the log-transformed total event count measured by FACS (also see Fig. 2C). B Correlation of the nasal sIgA concentration with the bacterial density expressed as the 16S rRNA gene copy number. Correlations were tested by linear regression analysis. [file 40168_2023_1675_MOESM3_ESM.pdf]

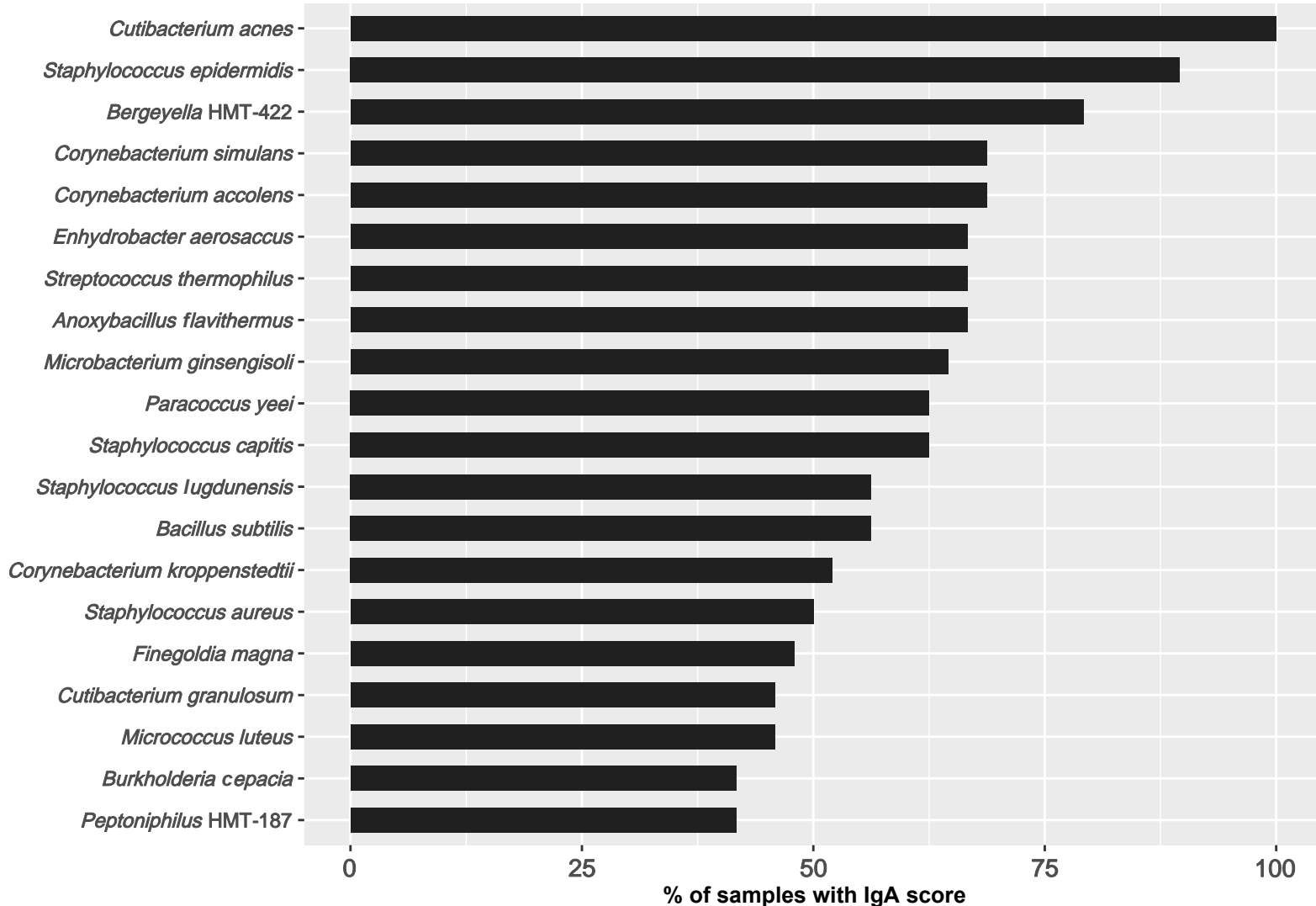

Supplement: Supplementary file 5 — Additional file 4: Figure S3. Top-20 most prevalent species, based on IgA scores. The frequency indicates in which percentage of the samples an IgA score for a given species could be calculated. [file 40168_2023_1675_MOESM4_ESM.pdf]

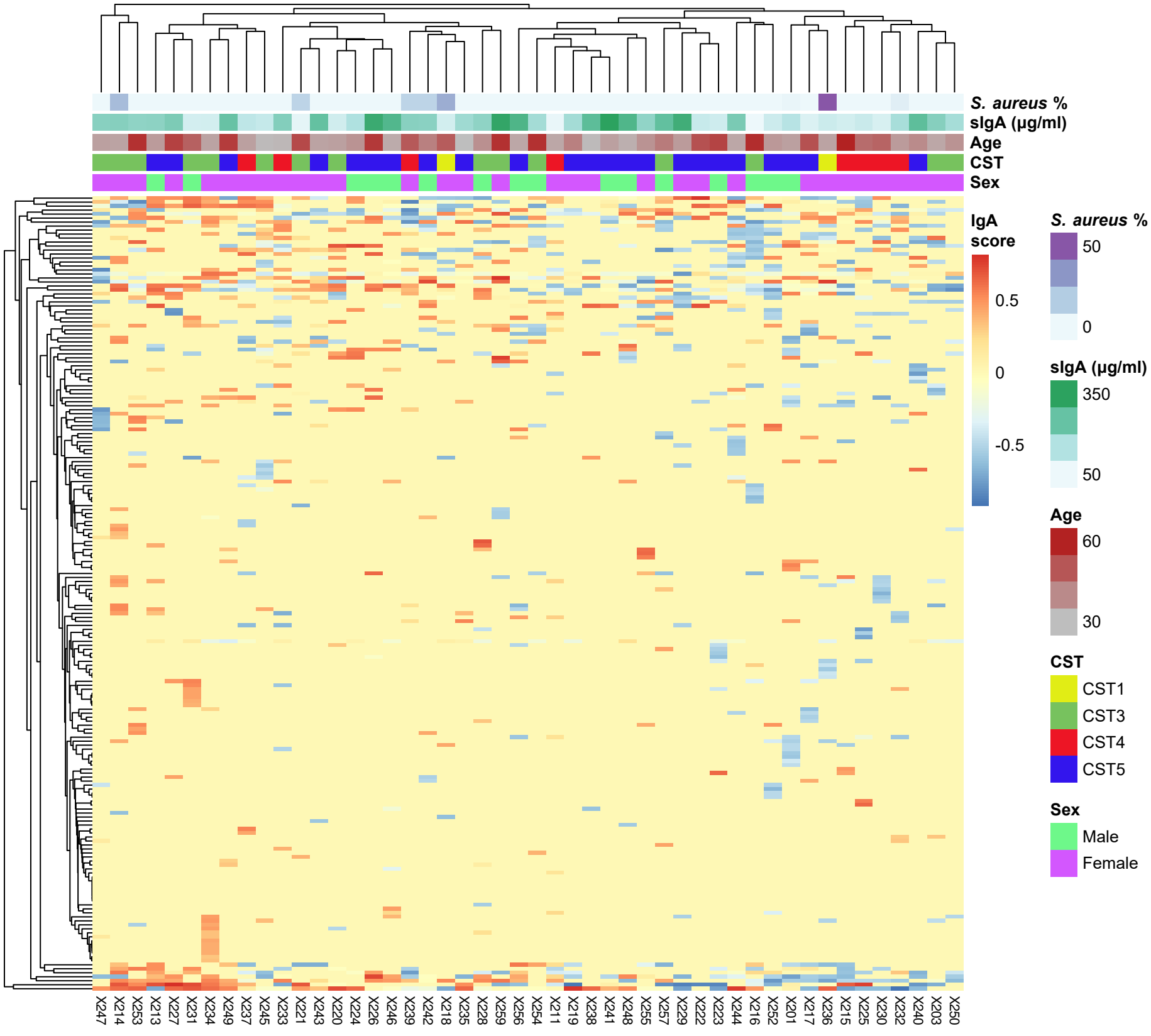

Supplement: Supplementary file 6 — Additional file 5: Figure S4. Hierarchical cluster analysis of the sIgA targeting. The study participants (columns) and the top-20 nasal bacterial species (rows) were clustered based on the IgA scores, with missing values (when no IgA score could be calculated due to absence of the species) treated as 0. The proportion of S. aureus reads, sIgA concentration and CST of the sample, as well as the age and sex of the respective study participant, are displayed at the top. Related to Fig. 4. [file 40168_2023_1675_MOESM5_ESM.pdf]
