# Supplementary material for: Secretory IgA impacts the microbiota density in the human nose
Source: Microbiome. 2023 Oct 21;11:233. doi: 10.1186/s40168-023-01675-y (PMC10589987; doi:10.1186/s40168-023-01675-y)
Supplement: Supplementary file 7 — Additional file 6: Table S1. Bacterial strains used in this study. [file 40168_2023_1675_MOESM6_ESM.docx]

### Table S1. Bacterial strains used in this study.

| **Species** | **Strain** | **Source** |
| --- | --- | --- |
| *Corynebacterium accolens* | 63VAs_B8 | Human nasal isolate [54] |
| *Corynebacterium simulans* | 50VAs_B5 | Human nasal isolate [54] |
| *Cutibacterium acnes* | Mü58 | Human nasal isolate [54] |
| *Escherichia coli* | IM08B | Cloning host for plasmid transfer to *S. aureus* [40] |
| *Staphylococcus aureus* | 35-1 | Human nasal isolate from in-house collection |
| *Staphylococcus aureus* | JE2 | Human abscess isolate, USA300 clone [55] |
| *Staphylococcus aureus* | JE2 SpA-AA | This study |
| *Staphylococcus epidermidis* | IVK83 | Human nasal isolate [5] |
| *Staphylococcus lugdunensis* | IVK28 | Human nasal isolate [56] |
