# Supplementary material for: Secretory IgA impacts the microbiota density in the human nose
Source: Microbiome. 2023 Oct 21;11:233. doi: 10.1186/s40168-023-01675-y (PMC10589987; doi:10.1186/s40168-023-01675-y)
Supplement: Supplementary file 8 — Additional file 7: Table S2. Primers used in this study. [file 40168_2023_1675_MOESM7_ESM.docx]

### Table S2. Primers used in this study.

| **Primer** | **Sequence (5’-3’)** | **Description** |
| --- | --- | --- |
| 518F | CAATTACCGCGGCTGCTGG | 16S rRNA gene V1-V3 amplification [29] |
| 27R | CCGAGTTTGATCMTGGCTCAG | 16S rRNA gene V1-V3 amplification [29] |
| 1 | CCTCACTAAAGGGAACAAAAGCTG | Amplification of SpA-AA gBlock 1 |
| 2 | GCGCGCGCAAGCTTTGGA | Amplification of SpA-AA gBlock 1 |
| 3 | GCGCGCGCAAGCTTAAAGG | Amplification of SpA-AA gBlock 2 |
| 4 | CGACTCACTATAGGGCGAATTG | Amplification of SpA-AA gBlock 2 |
| 5 | CCTCAGCACATAATGAACAACTTTCTA | PCR validation and Sanger sequencing *spa-AA* |
| 6 | CGTTCCTCTGGTCTGCGTTAAG | PCR validation, anneals to the introduced annealing site at residues 56-63 |
| 7 | CAACGTATATAAGTTAAAATTGGTTTGGA | Sanger sequencing *spa-AA* |
| 8 | GCAATACATAATTCGTTATATTATGATGAC | Sanger sequencing *spa-AA* |
| 9 | ACTGTATCACCAGGTTTAACGACA | Sanger sequencing *spa-AA* |
| 10 | CCTACGGGDGGCWGCA | 16S rRNA gene qPCR (BactQuant) [30] |
| 11 | GGACTACHVGGGTMTCTAATC | 16S rRNA gene qPCR (BactQuant) [30] |
| 12 | CTGAGACACGGTCCAGACT | Amplification of *S. aureus* 16S rRNA gene |
| 13 | GCACTCATCGTTTACGGCGT | Amplification of *S. aureus* 16S rRNA gene |
