# Supplementary material for: Secretory IgA impacts the microbiota density in the human nose
Source: Microbiome. 2023 Oct 21;11:233. doi: 10.1186/s40168-023-01675-y (PMC10589987; doi:10.1186/s40168-023-01675-y)
Supplement: Supplementary file 9 — Additional file 8: Table S3. HiFi gBlocks used in this study. [file 40168_2023_1675_MOESM8_ESM.docx]

### Table S3. HiFi gBlocks used in this study.

| **SpA-AA gBlock 1 (5’-3’)** |
| --- |
| TACATCTTGTTTTTGAATAATATCTCTATTACGCAAGTGTGCTGTATTCTAAAGTGCACTTGTGTTTTCTATTTTTTAATAAAACCTCAGCACATAATGAACAACTTTCTATTTTCTCCTCACTAAAGGGAACAAAAGCTGGGTACCCCTCAGCACATTCAAAGCCCCACTTTATTCTTAAAAATATTTTTTAACTCATATGTATTAAACCGCTTTCATTATAAAAAATATCTCTATATTTTATCTGTTTTTATTAATCGAAATAGCGTGATTTTGCGGTTTTAAGCCTTTTACTTCCTGAATAAATCTTTCAGCAAAATATTTATTTTATAAGTTGTAAAACTTACCTTTAAATTTAATTATAAATATAGATTTTAGTATTGCAATACATAATTCGTTATATTATGATGACTTTACAAATACATACAGGGGGTATTAATTTGAAAAAGAAAAACATTTATTCAATTCGTAAACTAGGTGTAGGTATTGCATCTGTAACTTTAGGTACATTACTTATATCTGGTGGCGTAACACCTGCTGCAAATGCTGCGCAACACGATGAAGCTCAACAAAATGCTTTTTATCAAGTCTTAAATATGCCTAACCTTAACGCAGACCAGAGGAACGGATTTATCCAAAGCCTAAAGGCCGCTCCTAGCCAGAGTGCAAATGTACTTGGAGAGGCACAAAAACTTAATGACTCTCAAGCTCCAAAAGCTGATGCGCAACAAAATAACTTCAACAAAGATCAACAGAGCGCCTTCTATGAGATATTGAACATGCCTAACCTAAACGAAGCGCAACGTAACGGCTTCATTCAAAGTCTTAAAGCTGCCCCAAGTCAAAGCACTAACGTTTTAGGTGAAGCTAAAAAATTAAACGAATCTCAAGCACCGAAAGCTGATAACAATTTCAACAAAGAACAGCAAAATGCTTTCTATGAAATCTTGAATATGCCAAACTTGAACGAAGAACAACGCAATGGTTTCATCCAAAGCTTGCGCGCGC |
| **SpA-AA gBlock 2 (5’-3’)** |
| GCGCGCGCAAGCTTAAAGGCAGCACCTTCACAATCAGCTAACCTATTGTCAGAAGCTAAAAAGTTAAATGAATCTCAAGCACCGAAAGCGGATAATAAGTTTAATAAGGAGCAACAGAACGCATTTTACGAGATCTTACATTTACCTAACTTAAACGAAGAACAACGCAATGGTTTCATCCAAAGCCTAAAAGCTGCCCCAAGCCAAAGCGCTAACTTATTGGCAGAAGCAAAAAAGTTAAATGATGCTCAAGCACCAAAAGCTGACAACAAATTCAACAAAGAACAACAAAATGCTTTCTATGAAATTTTACATCTTCCAAATTTAACTGAAGAACAACGTAACGGCTTCATCCAAAGCCTTAAAGCCGCTCCTTCAGTGAGCAAAGAAATTTTAGCAGAAGCTAAAAAGCTAAACGACGCACAGGCACCAAAAGAAGAGGATAACAATAAACCAGGTAAGGAGGATAACAATAAGCCGGGCAAGGAAGACAATAATAAACCAGGTAAAGAAGACAATAACAAGCCTGGCAAAGAAGACGGTAATAAACCTGGAAAGGAGGATAATAAGAAGCCAGGAAAGGAGGATGGCAACAAGCCTGGTAAAGAAGACAACAAAAAACCTGGCAAGGAAGACGGCAACAAGCCAGGAAAAGAAGATGGCAACAAACCTGGTAAAGAAGATGGTAACGGAGTACATGTCGTTAAACCTGGTGATACAGTAAATGACATTGCAAAAGCAAACGGCACTACTGCTGACAAAATTGCTGCAGATAACAAATTAGCTGATAAAAACATGATCAAACCTGGTCAAGAACTTGTTGTTGATAAGAAGCAACCAGCAAACCATGCAGATGCTAACAAAGCTCAAGCATTACCAGAAACTGGTGAAGAAAATCCATTCATCGGTACAACTGTATTTGGTGGATTATCATTAGCCTTAGGTGCAGCGTTATTAGCTGGACGTCGTCGCGAACTATAAGAGCTCCAATTCGCCCTATAGTGAGTCG |
